# IgH Diversity In an Individual With Only One Million B Lymphocytes

**DOI:** 10.1155/1993/17249

**Published:** 1993

**Authors:** Ada Lee, Soléenne Desravines, Ellen Hsu

**Affiliations:** Department of Biology New York University New York New York 10003 USA

**Keywords:** Antibody repertoire, metamorphosis

## Abstract

Immunoglobulin sequences from an individual *Xenopus laevis* froglet were analyzed for combinatorial and junctional diversity. In an animal with about 10^6^ B lymphocytes, at least 26 out of the 56 V_H_1 genes available in a diploid genome were expressed, as were all J_H_ segments. Junctional diversity was similar to that observed in *Xenopus* tadpole sequences, that is, little or no N diversification was found and the recombination site sometimes occurred in a region of V/D or D/J homology. The froglet IgH diversity is further restricted by the elimination of D-gene participation through direct V to J joining. Of the six complementary-determining regions (CDR) contributing to the structure of the antigen-combining site, CDR3 is the most variable in sequence and structure. Froglet IgH CDR3 are restricted to both aspects. Compared to IgH sequences isolated from a 5-month-old adult, froglet CDR3 were, on the average, two codons shorter; overall, 58% of the froglet Ig sequences isolated carried CDR3 of ≤ 7 codons, compared to 30% of the adult sequences. In addition to being shorter, the tadpole/froglet CDR3 are less variable in sequence, as the absence of N regions also results in the V/D and D/J junctions being derived from germline elements. We therefore suggest that latent anti-adult specificities are not eliminated *in situ*, in the tadpole, but rather that such germline gene segments, singly or in their combinations thereof, that can potentially react to adult self-epitopes after metamorphosis have been counterselected during the course of evolution.


*Developmental Immunology*, 1993, Vol. 3, pp. 211–222
Reprints available directly from the publisher
Photocopying permitted by license only

© 1993 Harwood Academic Publishers GmbH
Printed in the United States of America

### IgH Diversity in an Individual with Only One Million B Lymphocytes

ADA LEE, SOLÉENNE DESRAVINES, and ELLEN HSU*

*Department of Biology, New York University, New York, New York 10003*

Immunoglobulin sequences from an individual *Xenopus laevis* froglet were analyzed for combinatorial and junctional diversity. In an animal with about $10^6$ B lymphocytes, at least 26 out of the 56 $V_H1$ genes available in a diploid genome were expressed, as were all $J_H$ segments. Junctional diversity was similar to that observed in *Xenopus* tadpole sequences, that is, little or no N diversification was found and the recombination site sometimes occurred in a region of V/D or D/J homology. The froglet IgH diversity is further restricted by the elimination of D-gene participation through direct V to J joining. Of the six complementary-determining regions (CDR) contributing to the structure of the antigen-combining site, CDR3 is the most variable in sequence and structure. Froglet IgH CDR3 are restricted to both aspects. Compared to IgH sequences isolated from a 5-month-old adult, froglet CDR3 were, on the average, two codons shorter; overall, 58% of the froglet Ig sequences isolated carried CDR3 of $\leq 7$ codons, compared to 30% of the adult sequences. In addition to being shorter, the tadpole/froglet CDR3 are less variable in sequence, as the absence of N regions also results in the V/D and D/J junctions being derived from germline elements. We therefore suggest that latent anti-adult specificities are not eliminated *in situ*, in the tadpole, but rather that such germline gene segments, singly or in their combinations thereof, that can potentially react to adult self-epitopes after metamorphosis have been counterselected during the course of evolution.

KEYWORDS: Antibody repertoire, metamorphosis.

### INTRODUCTION

Of the six complementarity-determining regions (CDR) that contribute to the structure of the antibody-combining site, the most variable in sequence and length are the CDR3 of the heavy and light chains. This is true for all vertebrate Ig studied. Unlike CDR1 and CDR2, which are encoded by germline V gene segments, the sequences encoding CDR3 are somatically generated from the rearrangement of different gene segments. The heavy-chain CDR3 encompasses the region formed by the $V_H$/D junction, the D-gene segment, and the D/$J_H$ junction; in addition to combinatorial diversity resulting from the many available $V_H$, D, and $J_H$ segments, the site of joining is imprecise. Nucleotides can be either deleted from or added to the ends of the gene segments, the additions being of two types, N and P (Alt and Baltimore, 1982; Tonegawa, 1983; Lafaille et al., 1989).

However, during ontogeny, the processes resulting in CDR3 diversification seem to be partially restricted. In fetal or newborn mice and *Xenopus laevis* tadpoles, Ig sequences seem to have little N-region addition, and the site of recombination in $V_H$/D and D/$J_H$ is frequently in a region of sequence homology (Feeney, 1990; Gu et al., 1990; Bangs et al., 1991; Schwager et al., 1991). Both of these factors contribute to the formation of an antibody repertoire that is much less diverse than it is in adults. The biological significance of these restrictions is not clear.

Larval antibody-response characteristics are retained in animals inhibited from metamorphosing by treatment with goitrogens (Hsu and Du Pasquier, 1984, 1992). Thus, the tadpole repertoire is dependent upon the developmental, not temporal, events. A switch from the larval to the

*Corresponding author.

adult-type antibody response occurs approximately at the time of metamorphosis (Hsu and Du Pasquier, 1992) and it was suggested that the new adult-specific cells arose at this time (Flajnik et al., 1987). A tadpole is immunocompetent by 2–3 weeks of age, and in the 7–8th week of larval life, it undergoes morphological changes and cell replacement in virtually every tissue, including lymphoid organs (Du Pasquier and Weiss, 1973). By the end of the metamorphosis, it is a different animal, both in morphological appearance and in the genes it expresses. Although the tadpole is basically immunocompetent through metamorphosis, there is some evidence of suppression for T-cell but not B-cell function (Chardonnens and Du Pasquier, 1973; Du Pasquier and Bernard, 1980). We suggest that the tadpole antibody repertoire is limited so as not to create autoimmune problems at metamorphosis, when novel self-antigens appear. The antibody studies suggest that the tadpole repertoire is different from the adult one, in that different and fewer antibodies are produced by isogenic tadpoles to the same antigens (Du Pasquier et al., 1979; Hsu and Du Pasquier, 1984).

Here we report an analysis of the Ig sequences present in a froglet, 5 days past the completion of metamorphosis. Our motivation was not only to study the kind of junctional events present in animals of this interesting period, but also to examine the extent of diversity existing in immunocompetent froglets possessing only about $10^6$ B lymphocytes.

### RESULTS

Three separate batches of cDNA ("a", "b", and "c" series in Fig. 1) were prepared from liver RNA of a single *X. laevis* froglet. PCR amplifications and cloning of DNA fragments were carried out as described in the Materials and Methods section. One cDNA batch and one PCR amplification were prepared using the adult liver and spleen RNA. The nucleotide sequence at the VDJ junctions are shown in Figs. 1 and 2.

### Analysis and Comparison of Junctional Sequences of Froglet and Adult Ig

The probable 3' terminus of any particular $V_H1$ gene was decided by comparison with 24 *Xenopus* germline $V_H1$ sequences (Schwager et al., 1989; Wilson et al., 1992b). As only one *Xenopus* germline D sequence has so far been reported (Schwager et al., 1991), it is not possible to identify definitively D elements and locate their 5' and 3' ends. Instead, D sequences in this study were determined by comparison to consensus CDR3 motifs deduced from the large number of published *Xenopus* Ig cDNA sequences (Schwager et al., 1988, 1991; Haire et al., 1990; Hsu et al., 1989). The 5' ends of $J_H$ were determined after comparison with the germline gene sequences (Schwager et al., 1991). Potential N or P additions were identified by inspection.

Following Kabat et al. (1991), we consider CDR3 of the heavy chain to begin at the third residue after cysteine-92 and ending on the residue before tryptophan-103. Although the assignment of P or N nucleotides here is somewhat arbitrary because the particular $V_H$ or D germline sequences are not known, all the *Xenopus* sequences were analyzed the same way. These results are presented in Figs. 1 and 2.

There is little N-region addition in the froglet sequences compared to those of the adult. It is possible to determine the $J_H$ contribution by comparing the CDR3 sequence with the germline $J_H$ segment. There is little difference in the average number (±S.D.) of nucleotides deleted from the 5' end of $J_H$: 3.6 (±2.8) in the adult compared to 4.0 (±2.5) to 4.6 (±2.4) in the froglet "a" series, depending on whether all the doubtful junctional nucleotides are assigned to D or to $J_H$, respectively.

In many sequences, two or three nucleotides at the $V_H$/D and D/$J_H$ junctions could have been contributed by either $V_H$ or D and either D or $J_H$, respectively. In Figs. 1 and 2, such nucleotides are capitalized. This feature is much more common in the froglet sequences (Fig. 1: a17, a30, a43, b4, a65, a96, b19, b76, etc.) than it is in the adult sequences (Fig. 2: 1.45, 1.47). In mice, Ig sequences lacking N-region addition from fetal or adult sources display the same characteristics. It has been suggested that this type of sequence overlap contributes to the recombination event.

Thus, the features particular to fetal- and tadpole-derived Ig sequences, which distinguish them from adult sequences, are also present in the froglet sequences, even though the animal was sacrificed several days after the completion of metamorphosis.

| | V | N | D | N | | JH | CDR3 length | Group |
|---|---|---|---|---|---|---|---|---|
| a22x | gct aga gac | | tacgggggtggca | | actactttgactac | 3 | 10 | |
| a23x | gct aga | | tacgggggtggca | | ggtatttcgagcac | 8 | 9 | |
| a17 | gct | | ACgggggtggca | | gactac | 3 | 5 | |
| a51 | act aga | | Ggggtggca | | gcttac | 2 | 5 | |
| **a55[1]** | **gct aga** | | **Gggggtggcag** | | **ctggtatttcgagcac** | **8** | **9** | **1** |
| **c50[1]** | **gct aga** | | **Gggggtggcag** | | **ctggtatttcgagcac** | **8** | **9** | **1** |
| a30 | gct a | | tacggg | | GGTatttcgagcac | 8 | 6 | |
| **a44** | **gct aga gacatg** | | **tacggggg** | | **ctatgctttcgattac** | **5** | **10** | **2** |
| **c36** | **gct aga gacatg** | | **tacggggg** | | **ctatgctttcgattac** | **5** | **10** | **2** |
| a27 | aca agg | | tacgggggtggcag | | ctggttcgattac | 7 | 9 | |
| a43 | gct | | ACgggggtggcagc | | tgactac | 3 | 6 | |
| a70 | act ac | t | tacgggggtggcag | | ctggttcgattac | 7 | 9 | |
| a87 | gct aga | | tacgggggtgg | | Ctttgactac | 3 | 7 | |
| **a71[1]** | **gct aga gat** | | **tacgggggtgg** | | **Ctactttgactac** | **3** | **9** | **3** |
| **b64** | **gct aga gat** | | **tacgggggtgg** | | **Ctactttgactac** | **3** | **9** | **3** |
| **c64[1]** | **gct aga gat** | | **tacgggggtgg** | | **Ctactttgactac** | **3** | **9** | **3** |
| b4 | gct aga g | | ACgggggtggcag | | ctggtatttcgagcac | 8 | 10 | |
| **b12[1]** | **act aga tac** | | **tacgggggtggcag** | | **ctatgctttcgattac** | **5** | **11** | **4** |
| **c57** | **act aga tac** | | **tacgggggtggcag** | | **ctattctttcgattac** | **5** | **11** | **4** |
| b71 | gct aga c | | acgggggtggcag | | ctatgctttcgattac | 5 | 10 | |
| b88 | gct aga ga | | gggtggcag | | ctggttcgattac | 7 | 8 | |
| b16 | act aga | | gggggtggca | | attacttcgcttac | 2 | 8 | |
| b39 | act aga gat | | gggggtggcag | | tgctttcgattac | 5 | 9 | |
| c38 | gct aga g | | ACggggg | | ctttgactac | 3 | 6 | |
| c54 | act aga g | | ACgggggtggcag | | ctttgactac | 3 | 8 | |
| c55 | act aga | | tacgggggtggcagc | | tatttcgagcac | 8 | 9 | |
| c76 | gct | | acgggggtggc | | tgctttcgattac | 5 | 7 | |
| c109 | gct aca gaagc | | acggggg | c | ttgactac | 3 | 7 | |
| c110 | act aga gaa | | gggggtggcag | | ctggttcgattac | 7 | 9 | |
| c113 | gct aga cac | gg | cgggggtgg | | ctttgcttac | 2 | 8 | |
| | | | | | | | | |
| a4x[1] | gct gca g | gg | ctggggtggag | | ctggtatttcgagcac | 8 | 10 | |
| a62 | act aga gag | ca | ctggggtggagc | | ctac | 3 | 7 | |
| a72 | gca | | ctgggg | | Tttgactac | 3 | 4 | |
| b3 | gct aga gatg | | ggggtgg | | ctactttgactac | 3 | 8 | |
| b27 | gct aga gaca | | ctggggtgg | | actactttgactac | 3 | 9 | |
| | | | | | | | | |
| a53 | act aga taca | | gggtacagcta | t | ctttcgattac | 5 | 9 | |
| a65 | tct aga | | TACgctagcgggtac | | gctttcgattac | 5 | 9 | |
| a73 | gct aga g | | ACgctagcgggtacagc | | gactac | 3 | 8 | |
| a18x | gct aga g | | ACgctagcgggtacag | | CTActttgactac | 3 | 10 | |
| a21 | gct aga | | ctagcgggtaca | | GCTttcgattac | 5 | 8 | |
| **a96** | **gct aga** | | **gctagcggg** | | **TACtggtatttcgagcac** | **8** | **9** | **5** |
| **c42** | **act aga** | | **gctagcggg** | | **TACtggtatttcgagcac** | **8** | **9** | **5** |
| a82 | | | GCTAGcggg | | TACtatgctttcgattac | 5 | 7 | |
| b19 | gct aga g | | ACgctagcgggtacag | | CTAtgctttcgattac | 5 | 11 | |
| b78 | gct aga ca | g | tacgctagcgggtacag | | CTAtgctttcgattac | 5 | 12 | |
| b76 | gct aga ga | | tagcggg | | TACtttgactac | 3 | 7 | |
| b46 | gct aga g | | acgctagcggg | | TACtttgactac | 3 | 8 | |
| b52 | gct aga gc | | cta | | tgtctatcac | 4 | 5 | |
| c83 | gct aga c | | acgctagcgggtacag | | CTggttcgattac | 7 | 10 | |
| c107 | act aga gag | cta | agcgggt | | Attactttgcttac | 2 | 9 | |
| c12 | act aga tac | | taca | tgg | ttgcttac | 2 | 6 | |
| | | | | | | | | |
| a5x | gct agc | | ttctggagtgggag | | ctttgcttac | 2 | 8 | |
| a6 | act aga ta | | ctggagtgggag | | tgctttcgattac | 5 | 9 | |

FIGURE 1. CDR3 sequences isolated from a newly metamorphosed froglet. One hundred sixteen sequences were isolated from three liver cDNA preparations (a, b, c) after PCR amplification using $\mu$ and $V_H1$ primers; 44/49 CDR3 were unique in series "a," 27/29 in "b," 34/38 in "c"; the sequences with repeats within the series are indicated by superscript and listed in what follows. Repeated sequences were found between series and these are grouped 1–11. The sequences are grouped according to D usage (Hsu et al., 1989; Schwager et al., 1991), as follows: $D_H10$; $D_H12$; $D_H1$; $D_H6$; $D_H$ "gggaac" (L. Du Pasquier, personal communication); $D_H15$ "tggtac" (Schwager et al., 1991, Wilson et al., 1992b); N region only or short D; and no D at all. Sequences that can be attributed to either $V_H$ or D and either D or $J_H$ are capitalized. P sequences are underlined; the underlined nucleotides in lines 19 and 22 within the J region are mutations in the $J_H5$ and $J_H2$ sequences, respectively. [1]Repeated sequences found: (a55,a63), (a71,a84,a80), (a4x,a33,a47), (c50,c51), (c64,c90), (b58,b12), (b59,b33), (c9,c34,c117). [2]These sequences can also belong to the next group.

| | | | | | | | | |
|---|---|---|---|---|---|---|---|---|
| b80 | gct aga gagc | | ttctggagtgggag | | tatgcttactttgacatc | 1 | 12 | |
| b34 | gct agt gaga | | ggagtgggag | | ctttgcttac | 2 | 8 | |
| c20 | act aga ga | | ctgga | | actttgactac | 3 | 6 | |
| a14 | act | | ggga | | ACTttgcttac | 2 | 4 | |
| a60 | gct aga gac | | ggga | | ACTttgactac | 3 | 6 | |
| **a81** | **act aga** | | **ggga** | | **ACTggtatttcgagcac** | **8** | **7** | **6** |
| **c5** | **act aga** | | **ggga** | | **ACTggtatttcgagcac** | **8** | **7** | **6** |
| **b35** | **gct aga** | | **ggga** | | **ACTttgactac** | **3** | **5** | **7** |
| **c2** | **act aga** | | **ggga** | | **ACTttgactac** | **3** | **5** | **7** |
| c21 | act aga gaa | | ggga | | ACaactggttcgattac | 7 | 7 | |
| **a38** | **gct aga** | | **ccgggaactact** | | **tttgactac** | **3** | **7** | **8** |
| **c32** | **gct aga** | | **ccgggaactact** | | **tttgactac** | **3** | **7** | **8** |
| c77 | gct t | | ccggga | g | ctttgactac | 3 | 5 | |
| a90[2] | act aga gaca | | tgg | | actttgactac | 2 | 6 | |
| a50[2] | act aga ga | | tgg | | ctttgactac | 3 | 5 | |
| a74 | gct aga ca | ga | | | actttgactac | 3 | 5 | |
| **a91** | **gct aga catg** | **ca** | | | **aactggttcgattac** | **7** | **7** | **9** |
| **b9** | **gct aga catg** | **ca** | | | **aactggttcgattac** | **7** | **7** | **9** |
| **c111** | **gct aga catg** | **ca** | | | **aactggttcgattac** | **7** | **7** | **9** |
| a18 | gct aga | aa | | | ctttgactac | 3 | 4 | |
| b22 | gct agg | g | | | actatgctttcgattac | 5 | 6 | |
| b61 | act aga tata | c | | | tgctttcgattac | 5 | 6 | |
| b42 | gct agg | gggc | | | ttgactac | 3 | 4 | |
| c81 | gct aga cacatg | gg | | | ctttgactac | 3 | 6 | |
| c47 | act aga taca | ac | | | gactac | 3 | 4 | |
| a5 | gct aga gacac | | | | ctttgactac | 3 | 5 | |
| a22 | gct agg gaacg | | | | tgactac | 3 | 4 | |
| a49 | act agg a | | | | actttgactac | 3 | 4 | |
| a52 | gct | | | | aactggttcgattac | 7 | 4 | |
| a66 | gct aga g | | | | ACtggttcgattac | 7 | 5 | |
| a32 | act a | | | | actactttgactac | 3 | 4 | |
| a83 | act aga taca | | | | actttgactac | 3 | 5 | |
| **a86** | **act aga gaagc** | | | | **tgtctatcac** | **4** | **5** | **10** |
| **b59[1]** | **act aga gaagc** | | | | **tgtctatcac** | **4** | **5** | **10** |
| **c9[1]** | **act aga gaagc** | | | | **tgtctatcac** | **4** | **5** | **10** |
| a89 | gct aca gaag | | | | actttgactac | 3 | 5 | |
| a94 | act aga g | | | | ACtggtatttcgagcac | 8 | 6 | |
| a95 | tct aga t | | | | ACtggttcgattac | 7 | 5 | |
| **b2** | **gct aga g** | | | | **ACtttgactac** | **3** | **4** | **11** |
| **c3** | **gct aga g** | | | | **ACtttgactac** | **3** | **4** | **11** |
| b5 | act aga gaca | | | | ttgactac | 3 | 4 | |
| b23 | act ag | | | | ctttgactac | 3 | 3 | |
| b68 | gct aga gac | | | | gtctatcac | 4 | 4 | |
| b94 | gct aga g | | | | ACaactggttcgattac | 7 | 6 | |
| c14 | gct aga a | | | | ttgactac | 3 | 3 | |
| c59 | gct aga g | | | | ACtactttgactac | 3 | 5 | |
| c73 | act aga gaca | | | | actttgcttac | 2 | 5 | |
| c80 | gct aga ga | | | | gtactactttgactac | 3 | 6 | |
| c105 | gct ag | | | | tgctttcgattac | 5 | 4 | |
| c102 | gca aga g | | | | ACtttgactac | 3 | 4 | |
| c106 | gct aga gaca | | | | actttgactac | 3 | 5 | |
| c115 | act ac | | | | ctttgcttac | 2 | 3 | |
| | | | | | | | 6.8 ± 2 | |

FIGURE 1. CDR3 sequences isolated from a newly metamorphosed froglet. One hundred sixteen sequences were isolated from three liver cDNA preparations (a, b, c) after PCR amplification using $\mu$ and $V_H1$ primers; 44/49 CDR3 were unique in series "a," 27/29 in "b," 34/38 in "c"; the sequences with repeats within the series are indicated by superscript and listed in what follows. Repeated sequences were found between series and these are grouped 1–11. The sequences are grouped according to D usage (Hsu et al., 1989; Schwager et al., 1991), as follows: $D_H10$; $D_H12$; $D_H1$; $D_H6$; $D_H$ "gggaac" (L. Du Pasquier, personal communication); $D_H15$ "tggtac" (Schwager et al., 1991, Wilson et al., 1992b); N region only or short D; and no D at all. Sequences that can be attributed to either $V_H$ or D and either D or $J_H$ are capitalized. P sequences are underlined; the underlined nucleotides in lines 19 and 22 within the J region are mutations in the $J_H5$ and $J_H2$ sequences, respectively. [1]Repeated sequences found: (a55,a63), (a71,a84,a80), (a4x,a33,a47), (c50,c51), (c64,c90), (b58,b12), (b59,b33), (c9,c34,c117). [2]These sequences can also belong to the next group.

| | V | N | D | N | | JH | CDR3 length |
|---|---|---|---|---|---|---|---|
| 1.3 | gct aga gacatg | c | cgggggtggc | ggcaa | actttgactac | 3 | 11 |
| 1.12 | act agc gaccgg | | acgggg | | gcttactttgacatc | 1 | 9 |
| 1.25 | gct agg aagctt | | acgggggtggca | | gactttgcttac | 2 | 10 |
| 1.32 | gct aga gacat | | cgggggtgg | g | aactggtatttcgatcac | 8 | 11 |
| 1.40 | act aga gg | | gggggtgg | | atgcttactttgacatc | 1 | 9 |
| 1.45 | act aca g | | ACgggggtggcagc | tt | tgactttgcttac | 2 | 10 |
| 1.39 | gct aga c | ttg | cgggggt | c | tactatgctttcgattac | 5 | 10 |
| 1.19 | gct aga gacatg | g | tacggg | c | tgactttgcttac | 2 | 9 |
| | | | | | | | |
| 1.16 | gct aga gac | | atggggtggt | ta | acctggttcgattac | 7 | 10 |
| 1.24 | gct aga ag | cac | tgggg | gac | ttgcttac | 2 | 7 |
| | | | | | | | |
| 1.7 | gct aga gacatg | | tacgctagcgggtaca | | atgactttgcttac | 2 | 12 |
| 1.29 | act aga g | | agcgggtacagc | | atttcgatcac | 8 | 8 |
| 1.42 | gct aca gaag | ca | acgctagcgggtacag | | ggtatttcgagcac | 8 | 12 |
| 1.47 | gct aga g | | ACgctagcgggtac | cct | gactac | 7 | 8 |
| 1.34[1] | gct aga gaacatg | | gtac | g | gactac | 3 | 6 |
| | | | | | | | |
| 1.4 | gct tcc | | ttctggagtgggagc | tt | tgctttcgattac | 5 | 10 |
| 1.31 | gct aga gacat | | ttctggagtgggagc | cct | ctttgactac | 3 | 11 |
| | | | | | | | |
| 1.6 | gct aga cggcc | | tatggaggtagc | cct | ctatgctttcgattac | 5 | 12 |
| 1.17 | gct aga catg | | atggaggtagc | gg | ttactttgcttac | 2 | 10 |
| | | | | | | | |
| 1.35 | gct aga gatc | | gggxxgggag | | ctactatgcttactttgacatc | 1 | 12 |
| 1.49 | gct aga gagagagag | | ggggc | | tgctttcgattac | x | 9 |
| | | | | | | | |
| 1.5 | gct aga gatc | ag | | | tggttcgattac | 7 | 6 |
| 1.20 | act aga g | cacc | | | ctttgcttac | 2 | 5 |
| | | | | | | | |
| 1.14 | gct ggg a | | | | actttgactac | 3 | 4 |
| 1.18 | act ccc | | | | tttgactac | 3 | 3 |
| 1.30 | gcc cgg a | | | | actttgactac | 3 | 4 |
| 1.48 | gct aga gac | | | | gtctatcac | 4 | 4 |
| | | | | | | | 8.6 ± 3 |

FIGURE 2. CDR3 sequences isolated from a 5-month-old young LG15 adult. Twenty-seven sequences were isolated from spleen and liver cDNA after PCR amplification using $\mu$ and $V_H1$ primers. The sequences are grouped according to D usage (Hsu et al., 1989; Schwager et al., 1991), as follows: $D_H10$; $D_H12$; $D_H1$; $D_H6$; $D_H7$; $D_H$ unknown; N region only or short D; and no D at all. See legend to Fig. 1; underlined nucleotide in the J region of line 14 denotes mutation from a known germline sequence. [1]See footnote 2 in legend to Fig. 1. No repeated sequences were found.

### $V_H$, D, $J_H$ Usage

In the 91 unique CDR3 sequences isolated from the individual froglet, 6 different D and 7 different $J_H$ segments were used. This is as diverse a representation as the 7 D and 7 $J_H$ segments found in the 27 adult sequences. $D_H1$ and $D_H10$ were the most frequently appearing D elements in both sets of sequences. Although one reading frame seems to predominate, all three are used in the froglet. The sample size is too small to determine whether reading-frame usage differs from that in adults. There is no indication of D-D fusion or inversion in any of the sequences. In the froglet, $J_H3$ seems to be the most frequently used $J_H$ segment; this is not the case in the adult sequences isolated in this study, although it has been observed elsewhere in cDNA sequences originating from adult organs (Schwager et al., 1991).

Figure 3 shows some froglet CDR2 sequences. We based the determination of a unique $V_H$ on the most variable portion of the $V_H$ germline sequence, CDR2. Of 29 different "a" series CDR2 sequences determined, 3 sets of sequences contained a single base difference (a38/a49 and a33, a74/a91/a94 and a82, a55/a44/a60/a63 and a52). The observed rate of Taq mutations in the $\mu$ constant region in this study was found to be one change in 820 bases, or 0.12%, which is consistent with the overall error frequency of 0.25% reported by Saiki et al. (1988), so it is possible that these three differences in 2646 bases (codons

47–63) were generated *in vitro*. Although some members of one CDR2 group contain different CDR1 sequences (data not shown), they need not all be different $V_H$ genes. Although different germline *Xenopus* $V_H$ genes can indeed have common CDR1 or CDR2 (Schwager et al., 1989), the PCR artifact of *in vitro* recombination is well documented (Meyerhans et al., 1990) and was also found in this study. For example, clones a80, a84, and a71 share CDR3 but differ in CDR2; given the considerable diversity found at the IgH junctions, this finding is more likely to be due to *in vitro* recombination than to independent rearrangement events producing identical CDR3.

The constant-region sequences contain only two $\mu$ alleles, differing at bases 12 and 13 (cc or at). Thus, this individual was heterozygous, as are most outbred frogs (Hsu et al., 1989). We obtained a minimum estimate of 26 different $V_H1$ genes represented in the froglet pool. The $V_H1$ family consists of at least 28 $V_H$ elements per haploid genome (Wilson et al., 1992b), with a pseudogene frequency of < 15% (Schwager et al., 1989). That is, in this individual, at least half of the available $V_H1$ gene pool was expressed by the froglet.

### Direct V to $J_H$ Joining

In many froglet sequences, there were very few nucleotides between $V_H$ and $J_H$ (last group, Fig. 1). Most of these were probably the result of direct $V_H$ to $J_H$ joining rather than excessive nibbling back at the junctions. Because a nonamer-like sequence is located within the 23-bp spacer of the 5' recombination signal sequences of $J_H3$, $J_H4$, and $J_H7$ in *X. gilli* and $J_H3$ and $J_H7$ in *X. laevis* (Schwager et al., 1991), direct $V_H$-$J_H$ joining is possible in *Xenopus*. In accordance with the 12/23-bp rules, it should be possible for either $V_H$ or D to join to these particular $J_H$ segments, and, indeed, the majority of such short CDR3 sequences in Fig. 1 (24/27) and published elsewhere (Schwager et al., 1991) do so. Although

```

Clones          Positions a series        47 48 49 50 51 52 a  b  53 54 55 56 57 58 59 60 61 62 63 64 65

                |  |  |  |  |  |  |  |  |  |  |  |  |  |  |  |  |  |  |  |  |

70              tgggtgtctgctattagc---aatggtggggttagcacatactatgctgattcagttaaagga

4x,47           .........cg...atat---g..---..aag..a..t.a.......................

73,23x          .....a.g.caa......cctg....ga.tag..a..t.........................

18,32           ..................agtgg.a.c...ag...............................

18x,21,30,90    .........cg...a...---.c.ac....ga...............................

22x,5,80,84     .........cg.......---.gc.ac....g......................t........

72              .........cg...a...---.gx.ac....g...............................

17,87           ................aa---gg..a....ag...............................

14              .c.......ct...a...---.gc.ac....g........................g......

38,49           ..........a.......---.gc.a....ag...............................

33              ..........a.....a.---.gc.a....ag...............................

74,91,94        ..................---g.........g...............................

51              .........cg...a...---.gc.a.....g..c..t.........................

66              .........cg...a...---gxxxxc....g.....t.gt......................

82              ..................---gg........g...............................

83              .........ag...a...---.gc.ac....g.......gt......................

27              .....a...a......c.---.g..a....ag............ca.................

86              ..........t.......---.gc.a.....g..c..t.........................

89              .........cg.......---g.........g.......gt......................

96              ..................---gg...c....c.gag...........................

81              .........ta......a---gg..ac....g.....t.........................

50,71           ...t.....ag......a---gg..ac....g.....t.........................

55,44,60,63     ...t.a....a.....a.---.g..a....ag..ca...cg......................

52              ...t.a....a.....a.---tg..a....ag..ca...cg......................

62              ...t.a.g.ata...tat---cc.aa.....g..c..t....a....................

65              ...t.a....a.......---t...a....ag..ca...cg......................

6,22,43,53      ...t.agg..aa...ca.---cc.aa.....g..c....a.......................

95              ...t.a.g.cac...ca.---tc.---..a.ga.c............................

5x              ...t.a.g.caa...t..---cc..a....ag..c............................

```

FIGURE 3. CDR2 sequences from froglet $V_H1$ clones. The CDR2 sequences were determined for the "a" sequences shown in Fig. 1 and are grouped according to sequence identity. Dots indicate identity with the first sequence, which is clone a70, dashes indicate gaps, and x is undetermined. Codon positions are according to Kabat et al. (1991). CDR2 includes codons 50–65, but codons 47–49 of FR2 were heterogeneous and helped distinguish the clones.

such short CDR3 sequences are also present in the adult set, they are found more frequently among the froglet sequences.

### CDR3 Length Heterogeneity

The average number of CDR3 codons in the froglet sequences is 6.8±2, significantly shorter than the 8.6±3 codons found in the adult. The proportions of sequences containing shorter CDR3 in the population are very different: 53/91 (58%) of the froglet sequences contain CDR3 lengths of ≤ 7 codons, twice as frequent as in the adult (8/27, 30%).

In Fig. 4, the data obtained in this study were combined with the cDNA data from Schwager et al. (1991) in order to compare CDR3 lengths in tadpole/froglet with adult. Although the two sets overlap, the bulk of the tadpole/froglet sequences contain 5–9 codons in CDR3 compared to 8–11 codons in the adult. The longer CDR3 sequences in the adult are probably overall the result of an increase of one or two codons through N-region additions. Most of the short froglet sequences were from direct $V_H$ to $J_H$ joining. Thus, there is a shift in the antibody pool to longer CDR3 sequences in the course of development for the 3 months following completion of metamorphosis.

### Upper Limit for the Number of Different Rearrangements in the Froglet Lymphocytes

An upper limit for the antibody specificities existing in the froglet is the number of mature B cells it carried. In froglet of this stage, there are roughly $2\times10^5$ B cells from the spleen, thymus, and blood ($2$–$3\times10^5$ thymocytes of which < 4% are sIg+ (Du Pasquier and Weiss, 1973; Hsu et al., 1983), $2$–$3\times10^5$ splenocytes of which < 50% are sIg+ (Du Pasquier and Weiss, 1973), $5\times10^5$ sIg+ cell/ml blood), and 0.01–0.02 ml can be drawn from this size animal. For technical reasons, the number of B cells in the liver is difficult to determine. Considering that 30–40 times more total RNA is obtained from the liver of a froglet or metamorphosing tadpole (64–94 $\mu$g) than from the spleen (1.8–1.9 $\mu$g), and three to four times more liver RNA is needed to give the same $\mu$-signal intensity on a Northern blot (not shown), then the total amount of liver $\mu$ mRNA isolated from a froglet is about tenfold the amount from a spleen, or the equivalent of about $10^6$ B cells. This is an overestimate, as the recovery of RNA from pools of the tiny spleens is not the same as from the much larger livers. Therefore, it is safe to say that about $10^6$ B cells are an upper limit.

### Lower Limit for the Number of Different Rearrangements in Froglet Lymphocytes

The number of CDR3 sequences, i.e., $N$=91, is certainly a lower limit for the number of rearrangements involving the $V_H1$ family in the froglet. Among the three independent cDNA and PCR preparations (series "a"–"c"), $N_1$=80 CDR3 sequences that appeared only once, $N_2$=8 CDR3 sequences appearing in two series (Fig. 1: Groups 1, 2, 4, 5, 6, 7, 8, and 11), and $N_3$=3 CDR3 sequences appearing in all three (Fig. 1: Groups 3, 9 and 10). There must also be a substantial number of sequences, $N_0$, that were not recovered in any of the preparations. If each rearrangement had an equal chance of being recovered in each preparation, we could estimate $N_0$. Since the assumptions are not likely to be true, the calculation based on them gives us a lower limit for $N_0$ rather than an estimate. According to the well-known binomial distribution, $N_0/N=q^3$, $N_1/N=3pq^2$, $N_2/N=3p^2q$, where $p$ is the probability of recovery of any given clone in any given preparation, and $q=1-p$. These equations are solved to obtain $N_0=N_1^2/3N_2=(80\times80)/(3\times8)=267$. Thus, the lower limit for rearrangements to $V_H1$ segments is 267+91, or 358. We have analyzed only $V_H1$ sequences in this study; $V_H1$ is one of the three large families ($V_H1$–3), all of which contain about 20–30 gene segments in the *Xenopus* haploid genome (Hsu et al., 1989). Because all three families are expressed in the froglet liver (see Discussion), then the lower limit for $V_H$ rearrangements is 3× 358, or 1074.

### Are the Groups of Identical CDR3 Sequences Derived from a Single Rearrangement Event?

In deriving a lower limit for the number of rearrangements in the froglet, the sequences within each "Group" in Fig. 1 were assumed to be from a single rearrangement event. To justify this assumption, let us assume for the moment that it is not true in the case of Group 1, for example. Only 3 out of the 105 sequences share that particular V-D junction and 3/105 share that

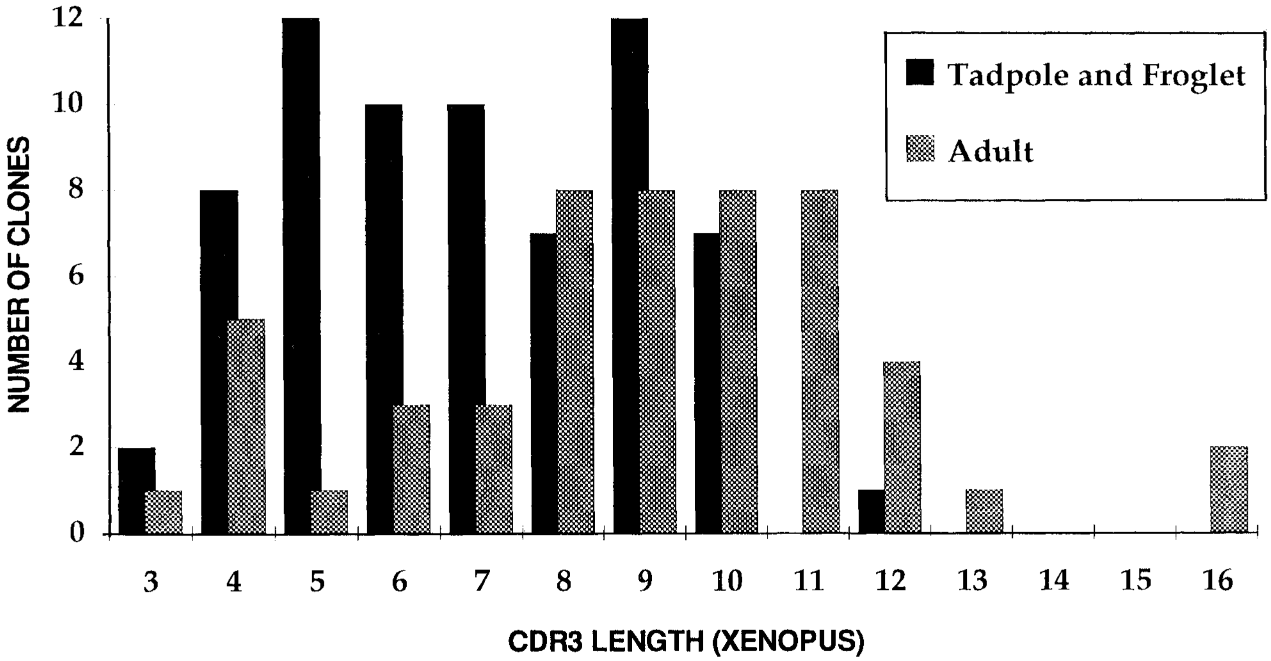

FIGURE 4. The lengths of CDR3 in young animals compared to adults. Distribution of *Xenopus* IgH CDR3 lengths from tadpoles (25 in-frame cDNA sequences taken from Fig. 4 in Schwager et al., 1991) combined with froglet (44 "a" series sequences from Fig. 1) and compared to those of the adult (25 in-frame cDNA sequences from Fig. 4 in Schwager et al., 1991, and 27 sequences from Fig. 2, this study).

particular D-J junction. The probability of occurrence of the Group 1 sequence by chance is then < 0.001. Thus, the original assumption is probably valid for almost all groups. Even if it were not, our lower limit is still valid: lowering $N_2$ and increasing $N_1$ simply increases the estimate of $N_0$.

### DISCUSSION

The *Xenopus* Ig heavy-chain gene system closely resembles those of mouse and man in organization and genetic complexity. Our finding of only two C$\mu$ alleles supports the suggestion that the 36-chromosome *Xenopus laevis*, thought to be a species derived by polyploidization from the 20-chromosome X. *tropicalis* (Bisbee et al., 1977), is diploidized at the IgH locus (Du Pasquier and Blomberg, 1982; Schwager et al., 1988). There are clusters of multiple $V_H$, D, and $J_H$ gene segments and few $C_H$ genes, which rearrange during B-lymphocyte differentiation to form a single transcription unit (Schwager et al., 1988). The number of gene segments is also comparable: there are 9 $J_H$ genes (Schwager et al., 1991), multiple D elements (Haire et al., 1989; Hsu et al., 1989; Schwager et al., 1991), and 3 large (20–30 genes) $V_H$ families along with at least 8 smaller $V_H$ families making up a total of > 80 unique $V_H$ genes per haploid genome (Haire et al., 1990; Hsu et al., 1989; Schwager et al., 1989). The combinatorial diversity could be great, as illustrated in these studies. Junctional diversity is increased by N and P additions, as well as usage of the D elements in inversion, D-D fusions, inversion-fusions, etc. (reviewed in Schwager et al., 1991).

Recent studies on immunoglobulin sequences expressed during development have shown that in fetal and newborn mice (Feeney, 1990; Gu et al., 1990; Bangs et al., 1991) and *Xenopus* tadpoles (Schwager et al., 1991), at least two factors affecting junctional diversity, N-region addition and the imprecision of the recombination sites, are more limited than in adults.

An individual froglet was chosen for these studies for several reasons. First, such an animal is in an interesting developmental stage, intermediate between tadpole and adult; we wished to examine the timing of the switchover to adult-type Ig assembly. Most of the Ig sequences obtained from the froglet had tadpole characteristics—sharing of sequence at the V/D

or D/J junctions and paucity of N-region addition. If the adult B-cell population was emerging at this time (Flajnick et al., 1987; Hsu and Du Pasquier, 1992), it contributed little to froglet liver RNA. This leads to a second point of interest: As suggested elsewhere (Flajnik et al., 1987), is the bulk of the larval B-cell population quickly eliminated at metamorphosis? The froglet Ig sequences are larvallike in nature, but we cannot be sure if these sequences were from newly differentiated adult B cells or from leftover tadpole B cells. If the latter, then antibodies from larval life must not be harmful to the metamorphosed froglet. If the former, then froglet $V_H$, D, and $J_H$ usage is similar to that of much older and larger adults.

Finally, all previous studies on Ig heterogeneity were based on pools. In this study, single animals were examined so that the extent of diversity in an individual could be assessed. The froglet carried on the order of one million B lymphocytes, which is the ceiling for the number of specificities if each clone was represented by one B cell. The minimum number of independent IgH rearrangements was calculated to be 1000. With the geometric mean of these two values as the best estimate for the extent of diversity, the animal carries an adequate enough repertoire to be able to mount an antibody response to a variety of injected antigens as DNP, TNP, human Ig light chain, and *Limulus* hemocyanin (Jurd et al., 1975; Du Pasquier et al., 1979).

The antibody specificities expressed by a froglet pool include at least the $V_H1$, $V_H2$, $V_H3$, and $V_H5$ families (obtained by PCR amplification; data not shown), so that members from the three largest *Xenopus* $V_H$ families as well as one small family are represented. Sequences from the $V_H1$ family were cloned and analyzed; different $V_H1$ genes were represented in the froglet Ig pool. Taking into consideration a pseudogene content of 15% (Schwager et al., 1989), it seems that about half of the 56 germline $V_H1$ genes in the froglet were expressed. There is no indication of biased $V_H$ gene usage, at least in the $V_H1$ family.

Although $J_H3$ is used often, the frequency is no higher than that found among published tadpole or adult cDNA clones (Schwager et al., 1991). Of the nine $J_H$ elements found in *Xenopus*, $J_H9$ is not present in *X. laevis* and $J_H6$ is thought to be nonfunctional because of its unusual sequence (Schwager et al., 1988, 1991). All other $J_H$ genes were expressed in the froglet. The most common D elements found were of the $D_H1$ and $D_H10$ groups (Hsu et al., 1989; Schwager et al., 1991), both of which were mostly in one reading frame, though examples of the other two frames were also isolated.

Although the degree of Ig gene combinatorial diversity seems to be comparable in tadpole/froglet and adult, the adult antibody response increases tenfold in affinity (Du Pasquier and Haimovich, 1976), compared to a complete lack of affinity maturation in *Xenopus* tadpoles (E. Hsu, unpublished observations). Adult antibodies can be much higher in affinity to the same antigen (Hsu and Du Pasquier, 1984, 1992), and are more heterogeneous (Du Pasquier et al., 1979). The lower diversity in froglet and tadpole IgH sequences could be due to the paucity of N- and P-region additions. Although the mechanisms by which N and P nucleotides are added is not known, the larval Ig pool carries heavy-chain CDR3 regions that are shorter than those of the adult. The CDR3 regions are also less variable in sequence, as the absence of N and P result in the V-D and D-J junctions being entirely derived from germline elements.

The range of adult CDR3 lengths tend to be one or two codons longer than tadpole (Fig. 4). In mammals, the same trend can be seen, but it is not as distinct as in *Xenopus*. Since CDR1 and CDR2 lengths generally do not tend to vary much among $V_H$ genes, especially within a family, the somatically generated CDR3 of varying sequence and lengths must contribute greatly to the structure of the antibody-combining site, for example, rendering it as a groove or a deep cavity (Padlan and Kabat, 1988). The actual influence of restricted CDR3 length on the repertoire is difficult to estimate, but it is well documented that for antigens such as phenyl oxazolone, dextran, galactan, progesterone (Berek and Milstein, 1988; Sawada et al., 1991), the length of mammalian heavy-chain CDR3 is a critical factor determining specificity.

From X-ray crystallography studies of three lysozyme-antibody complexes, it can be observed that the number of antigen contacts with IgH CDR3 decreased with the decreasing length of CDR3, while the number of contact sites with CDR1 and CDR2 increased in parallel (Amit et al., 1986; Sheriff et al., 1987; Padlan et al., 1989; Kabat and Wu, 1991). If these observations can be generalized, the antibody repertoire in the tadpole/froglet must be biased toward antigen recognition by CDR1 and CDR2, or, in other words, germline $V_H$ sequences. Furthermore, since N and P additions do not contribute significantly, CDR3 is also largely restricted to germline-encoded elements. IgH diversity is further limited by the elimination of the D genes by direct V to J joining. In contrast to adult CDR3 sequences, most of which carry N and P additions and are therefore dependent on other somatic processes, the larval antibody repertoire is mostly generated from germline sequences. We suggest that elimination of possible anti-adult antibodies does not take place *in situ*, in the tadpole, but has already occurred by selection of the germline Ig genes during the course of evolution.

In conclusion, IgH sequences isolated from a single froglet show combinatorial and junctional diversity at the DNA level, but CDR3 of the froglet tend to be shorter and much less heterogeneous than the adult. Because CDR3 is a very important structural determinant for the combining-site conformation, the combining sites from the Ig of young animals may be less variable in sequence and in dimensions. One may further speculate that, at least in *Xenopus*, short CDR3 are important in the earlier life of the animal. Three of 8 $J_H$ genes carrying the option of bypassing D entirely are unlikely to have arisen without selective pressures.

### MATERIALS AND METHODS

### Animals

*Xenopus laevis* tadpoles were purchased from Nasco, and their developmental stages were determined according to the morphological criteria of Nieuwkoop and Faber (1967) at the time of sacrifice; stage 66 is reached with the complete resorption of the tail. The 5-month-old *Xenopus laevis–Xenopus gilli* interspecies hybrids (LG15 strain) were a gift of Dr. Louis Du Pasquier; these animals had metamorphosed after 2 months and had been young adults for 3 months.

### cDNA Preparations and PCR Amplification

Total RNA was prepared from the individual livers of the *Xenopus* tadpoles and froglets and from the spleen and liver of the adults, as described (Wilson et al., 1992b). First-strand cDNA was synthesized in a 20-$\mu$l reaction containing 10 mM Tris HCl, pH 8.3, 50 mM KCl, 2.5 mM $MgCl_2$, 500 $\mu$M each dNTP, 12.5 mM 2-mercaptoethanol, 20 u RNAse inhibitor (Boehringer-Mannheim), 100 u SuperScript reverse transcriptase (BRL), 1–3 $\mu$g RNA and a *Xenopus* $\mu$ oligonucleotide (5' gaattcgactgggtccatagactc 3') as primer (prepared by M. Drillings, Howard Hughes Medical Institute, Protein Chemistry Core Facility). Two microliters of the cDNA preparation were added to a 50–100-$\mu$l reaction mixture containing 100 pmoles each of the $\mu$ oligomer and an oligomer containing sequence from FR1 of the $V_H1$ family (Wilson et al., 1992a) (5' gaattcgatgtacaacttgaccag 3') in Perkin Elmer Geneamp PCR buffer with 200 $\mu$M each dNTP and 2.5 u Amplitaq DNA polymerase (Perkin-Elmer-Cetus). The samples were subjected to 35–40 cycles of amplification consisting of 1 min at 94°C, 2 min at 55°C, and 2 min at 72°C. The PCR products thus generated were stained with ethidium bromide after electrophoresis in 1.5% agarose and appeared as a band of approximately 450 bp. The DNA was isolated using Geneclean (Bio 101) and dissolved in 10 mM Tris, pH 7.6; 1 mM EDTA, pH 8.

### DNA Cloning and Sequencing

The DNA fragments generated using $V_H1$ and $\mu$ oligomers from the unimmunized LG15 adult and from one *X. laevis* froglet, 5 days past stage 66 (end of metamorphosis), were treated successively with Klenow (USB), T4 DNA polymerase (NEB), and polynucleotide kinase (NEB) before cloning into the *Hinc*II site in pBluescript (Stratagene). Isolated DNA clones were sequenced by the dideoxy-chain termination method (Sanger et al., 1977) with Sequenase kits (USB) and $\mu$ or $V_H1$ primers. Sequences were aligned and determined by inspection.

### ACKNOWLEDGMENTS

We are greatly indebted, as usual, to Charles Steinberg and Louis Du Pasquier for their advice and suggestions. We also wish to thank Melanie Wilson and Elvin A. Kabat for their comments and for sharing unpublished data. This work was supported in part by BRSG Grant (NIH) 2 SO7RR07062-26 and NSF Grant MCB 9118753.

A.L. was supported by an NSF REU fellowship and S.D. was a participant of the Minority Opportunity Scholarship Training program.

*(Received March 2, 1993)*

*(Accepted March 23, 1993)*

### REFERENCES

Alt F.W., and Baltimore D. (1982). Joining of immunoglobulin heavy chain gene segments: Implications from a chromosome with evidence of three D-$J_H$ fusions. Proc. Natl. Acad. Sci. USA **79:** 4118–4122.

Amit A.G., Mariuzza R.A., Phillips S.E.V., and Poljak R.J. (1986). Three-dimensional structure of an antigen-antibody complex at 2.8 Å resolution. Science **233:** 747–753.

Bangs L., Sanz I.E., and Teale J. (1991). Comparison of D, $J_H$ and junctional diversity in the fetal, adult, and aged B cell repertoires. J. Immunol. **146:** 1996–2004.

Berek C., and Milstein C. (1988). The dynamic nature of the antibody repertoire. Immunol. Rev. **105:** 5–26.

Bisbee C.S., Baker M.A., Hadji-Azimi I., and Fischberg M. (1977). Albumin phylogeny for clawed frogs *(Xenopus)*. Science **195:** 785.

Chardonnens X., and Du Pasquier L. (1973). Induction of skin allograft tolerance during metamorphosis of the toad *Xenopus laevis*: A possible model for studying generation of self tolerance to histocompatibility antigens. Eur. J. Immunol. **3:** 569–573.

Du Pasquier L., and Bernard C.C.A. (1980). Active suppression of the allogeneic histocompatibility reactions during the metamorphosis of the clawed toad *Xenopus*. Differentiation **16:** 1–7.

Du Pasquier L., and Blomberg B. (1982). The expression of antibody diversity in natural and laboratory-made polyploid individuals of the clawed toad *Xenopus*. Immunogenetics **15:** 251–260.

Du Pasquier L., Blomberg B., and Bernard C.C.A. (1979). Ontogeny of immunity in amphibians: Changes in antibody repertoires and appearance of adult major histocompatibility antigens in *Xenopus*. Eur. J. Immunol. **9:** 900–906.

Du Pasquier L., and Haimovich J. (1976). The antibody response during amphibian ontogeny. Immunogenetics **3:** 381–391.

Du Pasquier L., and Weiss N. (1973). The thymus during the ontogeny of the toad *Xenopus laevis:* Growth, membrane-bound immunoglobulins and mixed lymphocyte reaction. Eur. J. Immunol. **12:** 773–777.

Feeney A.J. (1990). Lack of N regions in fetal and newborn mouse immunoglobulin V-D-J junctional sequences. J. Exp. Med. **172:** 1377–1390.

Flajnik M.F., Hsu E., Kaufman J.F., and Du Pasquier L. (1987). Changes in the immune system during metamorphosis of *Xenopus*. Immunol. Today **8:** 58–64.

Gu H., Förster I., and Rajewsky K. (1990). Sequence homologies, N sequence insertion and $J_H$ gene utilization in the $V_HDJ_H$ joining: Implications for the joining mechanism and the ontogenetic timing of Ly1 B cell and B-CLL progenitor generation. EMBO J. **9:** 2133–2140.

Haire R.N., Amemiya C.T., Suzuki D., and Litman G.W. (1990). Eleven distinct $V_H$ families and additional patterns of sequence variation suggest a high degree of immunoglobulin gene complexity in a lower vertebrate, *Xenopus laevis*. J. Exp. Med. **171:** 1721–1737.

Hsu E., and Du Pasquier L. (1984). Ontogeny of the immune system in *Xenopus*. II. Antibody repertoire differences between larvae and adults. Differentiation **28:** 116–122.

Hsu E., and Du Pasquier L. (1992). Changes in the amphibian antibody repertoire are correlated with metamorphosis and not with age or size. Dev. Immunol. **2:** 1–6.

Hsu E., Julius M.H., and Du Pasquier L. (1983). Effector and regulator function in T and B lymphocytes in the amphibian *Xenopus*. Ann. Immunol. (Inst. Pasteur) **13D:** 277–292.

Hsu E., Schwager J., and Alt F.W. (1989). Evolution of immunoglobulin genes: $V_H$ families in the amphibian *Xenopus*. Proc. Natl. Acad. Sci. USA **86:** 8010–8014.

Jurd R.D., Davies L., and Stevenson G.T. (1975). Humoral antibodies to soluble antigens in larvae of *Xenopus laevis*. Comp. Biochem. Physiol. **50B:** 65–70.

Kabat E.A., and Wu T.T. (1991). Identical V region amino acid sequences and segments of sequences in antibodies of different specificities. J. Immunol. **147:** 1709–1719.

Kabat E.A., Wu T.T., Perry H.M., Gottesman K.S., and Foeller C. (1991). Sequences of proteins of immunological interest, 5th ed. (Washington, D.C.: U.S. Dept. of Health and Human Services).

Lafaille J.J., DeCloux A., Bonneville M., Takagaki Y., and Tonegawa S. (1989). Junctional sequences of T cell receptor genes: Implications for $\alpha\beta$ T cell lineages and for a novel intermediate of V-(D)-J joining. Cell **59:** 859–870.

Meyerhans A., Vartanian J.-P., and Wain-Hobson S. (1990). DNA recombination during PCR. Nuc. Acids Res. **18:** 1687–1691.

Nieuwkoop P.D., and Faber J. (1967). Normal table of *Xenopus laevis* (Daudin), 2nd ed. (Amsterdam: North Holland).

Padlan E.A., and Kabat E.A. (1988). Model-building study of the combining sites of two antibodies to $\alpha$(1-6) dextran. Proc. Natl. Acad. Sci. USA **85:** 6885–6889.

Padlan E.A., Silverton E.W., Sheriff S., Cohen G.H., Smith-Gill S.J., and Davies D.R. (1989). Structure of an antibody-antigen complex: Crystal structure of the HyHEL-10 Fab lysozyme complex. Proc. Natl. Acad. Sci. USA **86:** 5938–5942.

Saiki R., Gelfand R.H., Stoffel S., Scharf S.J., Higuchi R., Horn G.T., Mullis K.B., and Erlich H.A. (1988). Primer-directed enzymatic amplification of DNA with a thermostable DNA polymerase. Science **239:** 487–491.

Sanger F., Nicklen S., and Coulson A.R. (1977). DNA sequencing with chain-terminating inhibitors. Proc. Natl. Acad. Sci. USA **74:** 5463–5467.

Sawada J.-I., Mizusawa S., Terao T., Naito M., and Kurosawa Y. (1991). Molecular characterization of monoclonal anti-steroid antibodies: Primary structures of the variable regions of seven antibodies specific for 17$\alpha$-hydroxy-progesterone or 11-deoxycortisol and their pH-reactivity profiles. Mol. Immunol. **28:** 1063–1072.

Schwager J., Buerckert N., Courtet M., and Du Pasquier L. (1989). Genetic basis of the antibody repertoire in *Xenopus:* Analysis of the $V_H$ diversity. EMBO J. **8:** 2989–3001.

Schwager J., Buerckert N., Courtet M., and Du Pasquier L. (1991). The ontogeny of diversification at the immunoglobulin heavy chain locus in *Xenopus*. EMBO J. **10:** 2461–2470.

Schwager J., Grossberger D., and Du Pasquier L. (1988). Organization and rearrangement of immunoglobulin M genes in the amphibian *Xenopus*. EMBO J. **8:** 2409–2415.

Sheriff S., Silverton E.W., Padlan E.A., Cohen G.H., Smith-Gill S.J., Finzel B.C., and Davies D.R. (1987). Three-dimensional structure of an antibody-antigen complex. Proc. Natl. Acad. Sci. USA **84:** 8075–8079.

Tonegawa S. (1983). Somatic generation of antibody diversity. Nature **302:** 575–581.

Wilson M., Hsu E., Marcuz A., Courtet M., Du Pasquier L., and Steinberg C. (1992a). What limits affinity maturation of antibodies in *Xenopus*—the rate of somatic mutation or the ability to select mutants? EMBO J. **11:** 4337–4347.
Wilson M., Marcuz A., Courtet M., and Du Pasquier L. (1992b). Sequences of C$\mu$ and the $V_H1$ family in LG7, a clonable strain of *Xenopus*, homozygous for the immunoglobulin loci. Dev. Immunol., **3:** 13–24.

